# Effect of Dietary Microalgae on Growth Performance, Profiles of Amino and Fatty Acids, Antioxidant Status, and Meat Quality of Broiler Chickens

**DOI:** 10.3390/ani10050761

**Published:** 2020-04-27

**Authors:** Sabry El-Bahr, Saad Shousha, Ahmed Shehab, Wassem Khattab, Omar Ahmed-Farid, Islam Sabike, Osama El-Garhy, Ibrahim Albokhadaim, Khaled Albosadah

**Affiliations:** 1 Department of Biomedical Sciences, College of Veterinary Medicine, King Faisal University, Al-Ahsa 31982, Box 400, Saudi Arabia; smmohamed@kfu.edu.sa (S.S.); ialbokhadaim@kfu.edu.sa (I.A.); kbusadah@kfu.edu.sa (K.A.); 2 Department of Biochemistry, Faculty of Veterinary Medicine, Alexandria University, Alexandria 21526, Egypt; 3 Department of Physiology, Faculty of Veterinary Medicine, Benha University, Benha 13518, Egypt; 4 Department of Nutrition and Clinical Nutrition, Faculty of Veterinary Medicine, Benha University, Moshtohor, Qalioubia, Benha 13518, Egypt; ahmedshehab@fvtm.bu.edu.eg (A.S.); wasimhassan@fvtm.bu.edu.eg (W.K.); 5 Department of Physiology, National Organization for Drug Control and Research, Giza 12622, Egypt; ebntaimya@yahoo.com; 6 Department of Food Hygiene, Faculty of Veterinary Medicine, Benha University, Benha 13518, Egypt; islamsabek@fvtm.bu.edu.eg; 7 Department of Animal Production, Faculty of Agriculture, Benha University, Moshtohor, Qalioubia, Benha 13518, Egypt; osama.elsayed1977@yahoo.com

**Keywords:** *Chlorella vulgaris*, *Spirulina platensis*, *Amphora coffeaformis*, meat quality, biomarkers

## Abstract

**Simple Summary:**

The use of feed additives with no side effectsfor enhancing growth performance and improving meat quality in broilers chickens is an essential research topic. In these regards, this study aimed to evaluate the impact of three species of microalgae namely Chlorella vulgaris (CV), Spirulina platensis (SP) and Amphora coffeaformis (AC) on growth performance, profiles of fatty and amino acids, antioxidant status and meat quality of breast muscles. The results demonstrated that the inclusion of studied microalgae notably AC has a positive effect on performance, antioxidant status and meat quality of breast muscle in broiler chickens.

**Abstract:**

The study used 96 broiler chickens to evaluate the impact of three species of microalgae on performance, profiles of fatty and amino acids, antioxidants, and meat quality of breast muscles. Birds were divided into four groups (24 birds/each) with 4 replicates (6 birds each). Birds in the first group were fed basal diet and served as a control (C). Birds of 2–4 groups were fed basal diet mixed with same dose (1 g/kg diet) of *Chlorella vulgaris* (CV), *Spirulina platensis* (SP), and *Amphora coffeaformis* (AC). At the age of 36 days, performance parameters were reported, and breast muscle samples were collected and stored frozen at −80 °C. AC shared CV in the superiority of increasing final body weight and body weight gain compared to SP and control. AC shared SP in the superiority of increasing the level of essential fatty and amino acids and decreasing the microbial growth in breast muscle compared to CV and control. All studied microalgae reduced malondialdehyde (MDA) and protein carbonyl (PC) levels, cooking loss, and aerobic plate count (APC) and increased the superoxide dismutase (SOD) activities in breast muscle compared to control. The current study indicated that studied microalgae, notably AC, can be used to enhance performance and meat quality in broilers chickens.

## 1. Introduction

The rapid growth of chicken broilers, the availability of its high-quality meat at low sales prices, and its rapid response to changes in ration composition have increased the researchers attention to producing chicken meat enriched with functional ingredients [1]. Antibiotics have been widely used in diets of livestock for many years to control disease and improve production performance [2]. However, there is a global trend to reducing their use in poultry feeds to avoid the risk of antibiotic residues in meat and minimize the development of antibiotic resistance [3,4,5,6]. Therefore, the addition of antibiotics as growth promotersin animal diet has been prohibited [7] Hence, searching for potential growth promoting alternatives with no side effects is very essential [8]. Different kinds of phytogenic feed additives, especially marine plants and other natural materials, were used to improve animal health and production due to their anti-inflammatory, immunomdulatory, antioxidant, and antibacterial activities [9]. Microalgae are unicellular, photosynthetic, and microscopic algae live in marine and fresh water that contains several biological active components such as omega-3 (n-3) long chain polyunsaturated fatty acids (LC-PUFA), essential amino acids, antioxidants, and carotenoids [10]. During the last decade, the beneficial feeding effects of microalgae have been advertised extensively all over the world, making the algae production enterprises begin to spread among small and large producer’s levels [11]. *Spirulina platensis* (SP) is a prokaryotic cell type blue green algae and is some time called cyanobacteria [12]. Dried SP is a rich source of protein, essential fatty acids, vitamins and carotenoids, so it can be used for human and animal feeding [13]. *Chlorella vulgaris* (CV) is a green microalgae, unicellular live in freshwater and is a rich source of essential aminoacids, vitamins, minerals, antioxidants, and carotenoids [14]. The antibacterial, antioxidant, hypolipidemic, immunomodulatory, and anti-inflamatory effects of SP and CV have been studied in both laboratory animals [15] and livestock as well as poultry [16,17]. Recently, three weeks administration of SP for broilers resulted in similar or even better responses than administration of in-feed antibiotics during the whole rearing period [18]. *Amphora coffeaformis* (AC) exert potent antioxidant effect against lipid peroxidation [19], and rich in polyunsaturated fatty acids (PUFAs), especially α-linolenic acid, eicosapentaenoic (EPA), and docosahexaenoic (DHA) [20]. However, little literature is available about dietary supplementation of AC.

Microalgae improved the fatty acids profile but failed to improve the growth performance, carcass, traits, color, pH, oxidative stability, chemical composition, and sensory characteristics in Muscovy ducks [21]. On the contrary, microalgae, especially SP and CV, improved the growth performance and meat quality through enrichment of broiler meat with n-3 polyunsaturated fatty acids (PUFA) and carotenoids [12]. Furthermore, microalgae demonstrated antimicrobial activities against spoilage and foodborne microorganisms, therefore trials are still seeking to validate it as potential alternatives to traditional preservatives [22]. Many algal compounds including proteins, amino acids, PUFA, especially eicosapentaenoic (EPA), docosahexaenoic (DHA), polysaccharides, and antioxidants have demonstrated antimicrobial potential [23], but the primary algal components responsible for antimicrobial functionality are still a relatively incipient field of research [24]. Even so, comparative studies investigating the effect of different species of microalgae on different axis such as fatty and amino acids composition, antioxidant status, and meat quality parameters in broiler chicken meat are needed. Therefore, the current study was conducted to evaluate impact of dietary dried microalgae supplementation on the growth and meat quality parameters of broiler chickens.

## 2. Materials and Methods

### 2.1. Birds, Diets, and Experimental Design

Experimental procedures and management conditions used in this study were carried out in accordance with the national institute of health guidelines for the care and the use (NIH Publications No.8023, revised 1978) and approved by Animal Care and Use Committee of Benha University, Faculty of Veterinary Medicine, Egypt (BUFVTM; permission # 282019 at 02 August 2019). A total number of 96 one day old Cobb 500 broiler chicks (mixture of male and female) were purchased from certified Cobb breeder flock of hens (Benha, Egypt). The day-old chicks were transferred to Poultry research Farm, Faculty of Agriculture, Benha University, Egypt where the field experiment was conducted. At their 4th day of age, birds were weighed (73.54 ± 0.49) and then distributed in 16 pens (6 birds in a pen) at room temperature of 33 °C. Temperature was maintained at 33 °C during the first week and gradually decreased to 24 °C by the end of the third week until the end of the experiment. The relative humidity maintained at 50–60% during the experimental period. The air exchange range was 0.3 m^3^/minute/m^2^. Birds were exposed to 23 h of white light a day and 1 h a dark. The intensity of light was 30–40 Lux from 0–7 day of age, thereafter the intensity was 5–10 Lux until the end of the experiment. 

The pens provided a floor area of 1.5m^2^ for each pen. The regular vaccination program applied to all birds. The birds of all groups were vaccinated against Newcastle disease virus (Hitchner B1) at day 7 in drinking water, infectious bursal disease virus (Gumbo L strain) at day 14 in drinking water, and Newcastle disease virus (La Sota) at day 21 of age in drinking water. The basal diet was formulated to meet the nutrient requirements of broiler chickens [25]. The composition of basal ground feed diet is displayed in Table 1. Throughout the experimental period (from 4 to 36 days of life), clean water and feeds were supplied ad libitum for broiler chickens. Birds were divided into four groups (24 birds for each) with 4 replicates (6 birds each; 4 × 4 × 6). Birds in the first group were fed basal diet alone and served as a control (C). Birds of 2–4 groups were fed the basal diet supplemented with same dose (1 g/kg diet) of *Chlorella vulgaris* (CV), *Spirulina platensis* (SP), and *Amphora coffeaformis* (AC), for 32 days, respectively. The dried powder of the three microalgal species was obtained from the Algae Production Unit (APU), National Research Institute, Cairo, Egypt. The fatty and amino acids profiles of studied microalgae are illustrated at Table 2 and Table 3, respectively. Fatty acid methyl ester (FAME) were used for quantitative determination of free fatty acids using gas chromatography (GC; Agilent Technologies Inc., Wilmington, DE, USA) equipped with SP2330 column (30 mm × 0.32 mm × 0.2 μm film thickness; Supelco Analytical, Bellefonte, PA, USA) and flame ionization detector. FAME peaks identified by comparison with retention times of mixture of fatty acids standards (Cat. No. 24073, Sigma-Aldrich, St. Louis, MO, USA) using Hewlett-Packard ChemStation software (Agilent). Fatty acids values were expressed as mg/g dried powder [26,27,28]. Derivatized microalgae samples as well as amino acid standards (Sigma-Aldrich, St. Louis, MO, USA) were injected into the HPLC (Agilent HP 1200 series apparatus) equipped with Nova-PakTM C18 column (4 μm, 3.9 × 4.6 mm) for separation and quantification of free amino acids (mg/g dried powder) in studied microalgae [29].

### 2.2. Growth Performance

At the end of the experiment, performance parameters, namely body weight, body weight gain, and feed intake were estimated. Feed conversion ratio (FCR; kg feed/kg gain) was calculated by dividing feed intake with body weight [30].

### 2.3. Collection of the Breast Meat Samples

All chickens from each group were humanely euthanatized and samples from breast muscles (*Pectoralis major*) were dissected, cleaned, and stored frozen at −80 °C for analysis of profiles of amino and fatty acids, antioxidant status and meat quality. Euthanasia was performed after sodium pentobarbital anesthesia (20–30 mg/kg) [31]. Sodium pentobarbital was injected to wing vein with sterilized needles.

### 2.4. Determination of Profiles of Fatty and Amino Acids in Breast Muscles

Chloroform and methanol (2:1; v/v) solution was used to extract the total lipids from the breast muscle after vortexing for 2 min and centrifugation for 10 min at 1792 g [26]. The obtained supernatant was used to prepare the fatty acid methyl esters (FAME) by using methanol/sulphuric acid mixture (95:5) and hexane following esterification process as outlined earlier [28]. The obtained hexane extract of FAME were used for quantitative determination of free fatty acids using gas chromatography (GC; Agilent) equipped with SP2330 column (30 mm × 0.32 mm × 0.2 μm film thickness; Supelco Analytical, Bellefonte, PA, USA) and flame ionization detector [27]. FAME peaks identified by comparison with retention times of mixture of fatty acids standards (Cat. No. 24073, Sigma-Aldrich, St. Louis, MO, USA) using Hewlett-Packard ChemStation software (Agilent). Fatty acids values were expressed as µg/g meat tissue. Homogenized breast meat samples were prepared, centrifuged, and filtrated [28] and then the filtrate was derivatized [32]. Derivatized samples as well as amino acid standards (Sigma-Aldrich, St. Louis, MO, USA) were injected into the HPLC (Agilent HP 1200 series apparatus, USA) equipped with Nova-PakTM C18 column (4 μm, 3.9 × 4.6 mm) for separation and quantification of free amino acids (nmol/g meat) according to the method described earlier [29] with some modifications [28]. 

### 2.5. Determination of Antioxidant Status

Malondialdhyde (MDA) was measured using HPLC (Agilent HP 1200 series apparatus) following protocols outlined earlier [33,34]. Briefly, an ice-cold 0.1 M Tris (hydroxymethyl) aminomethane-HCl (Tris-HCl) was used to prepare a 10% muscle homogeneate (w/v) at pH 7.4 by using an ice-cold homogeniser (Glas-Col, Terre Haute, IN, USA). The homogenate was then centrifuged at 2000× g at 4 °C for 15 min to eliminate nucleus and debris. The stock MDA standard solution of 1 mM concentration was prepared by dissolving 25 μL of 1,1,3,3 tetraethoxypropane (TEP) in 100 mL of water. Then, a 20 nmol/mL working standard was prepared by hydrolysis, for 2 h at room temperature, of 1 mL of TEP stock solution in 50 mL of sulfuric acid (1%), which was again diluted with 1% sulfuric acid to achieve a final standard concentration of 1.25 nmol/mL for the assessment of the total MDA. Analytical column Supelcosil C18 (5 μm particle size and 80 Ao pore size) (250 × 4.6 ID), mobile phase 82.5:17.5 (v/v) of 30 mM monobasic potassium phosphate (pH 3.6)–methanol, a flow rate of 1.2 mL/min and a wavelength of 250 nm were used for HPLC detection. The spectrophotometric assay described earlier Castegna, et al. [35] for estimating protein carbonyl (PC) from muscle samples was performed. Muscle samples were extracted in tricholoroacetic acid (TCA) by a final concentration of 10% (w/v) for the derivatization step. The precipitates were then treated with 500 μL of 0.2% dinitrophenylhyrdazine (DNPH) and incubated at room temperature for 1 h at 5 min intervals with vortexing. A total of 55 μL of 100% TCA was added to precipitate the proteins. The subsequent pellets were centrifuged and washed three times with 500μL of ethanol:ethyl acetate mixture (1:1; v/v). The pellet was then dissolved in 600 μL of 6 M of guanidine hydrochloride. The absorbance was determined at wavelength of 370 nm against an appropriate blank. Breast muscle tissue homogenate was prepared, centrifuged (4000 rpm for 15 min at 4 °C) and the obtained supernatant was used to determine the superoxide dismutase (SOD) activity by using ELISA commercial kits (Cat # SD 25 21; Biodiagnostic Company, Giza, Egypt) according to the manufacturer’s instructions. 

### 2.6. Estimation of Meat Quality Parameters

The collected breast muscles were used to analyze the quality parameters namely, pH after 24 h (pH_24_), water holding capacity (WHC), thawing loss, and cooking loss. The pH_24_ of the collected breast muscles was recorded by using pH-meter (Jenway 3510 pH-meter, Cole-Parmer, Staffordshire, United Kingdom) 24 h post slaughtering. Three points calibration at 4, 7, and 10 pH was used in the current study. The range was −2.000 to 19.999 pH; resolution was 0.001/0.01/0.1 pH and the accuracy was ±0.003 pH. The low-speed centrifugation method was conducted to estimate WHC of breast muscles, with a little modification. Briefly, about 10 g of intact breast muscle was placed and centrifuged in falcon tube containing glass beads at 10,000× *g* and 5 °C for 20 min, then the precipitated meat was instantly removed, dried with filter paper, and reweighed again. The WHC was calculated as the percentage of loss in muscle samples weight after centrifugation [36]. Regarding thawing loss, the breast fillet was trimmed, wiped dry, then weighed (initial weight) and stored at –18 °C. After one week, the frozen breast fillets were thawed at 5 °C for 24 h and the final weight was calculated. The percentage of the difference between initial and final weight was the value of thawing loss [37]. Cooking loss was determined as described earlier [37]. Briefly, the muscle fillets were separately placed in thin-walled thermotolerant plastic bags in a water bath until core temperature reached 70 °C, after which they were cooled to 5 °C in crushed ice, and reweighed again to calculate the cooking loss. Total aerobic plate count (APC) was conducted on the first day and after five days of chilling to evaluate the effect of supplemented microalgae on the overall microbiological quality of breast muscles. Representative chicken fillets for each group were immediately dissected from the carcasses under aseptic condition and part was allocated to the APC at the first day, while the other part was placed separately in sterile plastic bottles and held at 5 °C for further determination of APC after 5 days. Meat samples were prepared in the same way as for natural microflora of beef [38]. Briefly, a 10 g of chicken fillet were homogenized with a 90 mL sterile peptone solution (0.1%) to make a 10% meat mixture. A serial 10-fold dilution of the samples homogenate was then prepared using sterile normal saline, and the dilutions of each sample were inoculated in duplicate into APC agar. The plates were then incubated at 37 °C for 24–48 h before colonies were counted [39]. Then, the difference between APC (5th day) and APC (1st day) of chilling was calculated for each group to evaluate the preservative effect of supplemented microalgae on breast muscles.

### 2.7. Statistical Analyses

The collected data were exposed to one-way Analysis of Variance (ANOVA) using SPSS (version 16; IBM, Chicago, IL, USA) followed by Duncan’s multiple comparison tests [40] to compare the differences between dietary treatments, where significant differences were observed (*p* < 0.05).

## 3. Results

### 3.1. Effect of Dietary Microalgae on Growth Performance Parameters

Data summarized in Table 4 indicated that the final body weight and body weight gain increased significantly (*p* < 0.05) in birds that received a diet supplemented with CV and AC compared to that of birds supplemented with SP which remained comparable to the control. However, the feed intake and feed conversion ratio (FCR) during the overall experimental period remained significantly unchanged (*p* > 0.05) in all experimental groups compared to the control (Table 4).

### 3.2. Effect of Dietary Microalgae on Profiles of Fatty and Amino Acids in Breast Muscles

Fatty acid composition of breast muscle tissue of broiler chicken fed different species of microalgae is listed in Table 5. Eicosapentaenoic (EPA), docosahexaenoic (DHA), arachidonic acid (AA), polyunsaturated fatty acids (PUFA), and total n-3 fatty acids (n-3 FA) were increased significantly (*p* < 0.001) in breast muscle of birds supplemented with either SP or AC compared to that in birds received CV which remained comparable to control (Table 5). The presented data (Table 5) indicated that, the other fatty acid concentrations, total saturated fatty acids (SFA), total monounsaturated fatty acids (MUFA), and PUFA/SFA ratios remained unchanged significantly (*p* > 0.05) in all experimental groups compared to the control.

The influence of dietary microalgae on the amino acid profile in the breast muscles is displayed in Table 6. The levels of essential amino acids (lysine, methionine, tryptophan, and histidine) and aspartic acid were significantly increased *(p* < 0.001) in breast muscle of broilers supplemented with either SP or AC compared to that in birds received CV which remained comparable to control (Table 6). Other amino acid concentrations remained unchanged significantly (*p* > 0.05) in all experimental groups compared to the control (Table 6).

### 3.3. Effect of Dietary Microalgae on Antioxidant Status of Breast Muscles

The effect of dietary microalgae on the levels of MDA, SOD, and PC is presented in Table 7. All studied microalgae species induced a significant reduction of malondialdehyde (MDA) and protein carbonyle (PC) levels with significant increase in superoxide dismutase (SOD) activities in the breast muscle of broiler chickens compared to the control significantly.

### 3.4. Effect of Dietary Microalgae on Meat Quality Parameters of Breast Muscles 

Data that explore the effect of dietary microalgae on meat quality parameters of breast muscles of broiler chickens are shown in Table 8. All studied microalgae reduced the cooking loss, and had low aerobic plate count after first day (APC 1st day) and after 5 days APC (5th days) (*p* < 0.05) compared to the control (Table 8). Further, the differences between initial and 5th day APCs in breast muscle of broilers fed SP and AC was significantly lower (*p* < 0.05) than CV supplemented group which remained comparable to the control (Table 8). Other meat quality parameters (i.e., pH, WHC, and thawing loss) remained unchanged significantly (*p* > 0.05) in all experimental groups compared to the control (Table 8).

## 4. Discussion

### 4.1. Effect of Dietary Microalgae on Growth Performance Parameters

The incorporation of microalgae in animal diet offers a chance to enhance growth performance and meat quality but the results extensively based on chemical constituent, level of inclusion, types of microalgae, and environmental conditions during growth [41,42]. In the current study, the effects of three species of microalgae (CV, SP, and AC) have been compared at five axis (growth promotion parameters, meat composition of fatty and amino acids, meat antioxidant status, and meat quality parameters) in broiler chickens. The Cobb 500 performance guide would result in an average body weight of 2369 g. However, in the current study, the control line reached an average body weight of 1844 g. This may be attributed to feeding these birds with basal diet of mash ground feed without any additives. The supplemented basal diet was prepared in the college laboratory and not prepared in the feed mill, so the feeds were not pelleted or crumbled. In addition, the basal diet was not treated with steam during processing. These deviations from Cobb 500 dietary recommendations may be the reasons that stand behind the observed lower body weight of the control birds in the current study compared to that described in the Cobb 500 performance guide. In the current study, AC shared CV in the superiority of increasing final body weight and body weight gain compared to SP which remained comparable to the control. This current finding supported by the previous published reports [14,43] in broilers chickens. Furthermore, addition of fermented CV in diets of duck was associated with positive increases in body weight gain [44]. In the current study, FCR remained comparable to the control whatever the microalgae added. On the contrary, lower FCR was observed in birds fed CV and AC compared to control in broilers [45] and layers [46]. These diverse results reflect the role of microalgae types and the level of inclusion. As explained above, it can be said that AC and CV were preferable to SP in growth promotion of broilers when all were compared to the control. 

### 4.2. Effect of Dietary Microalgae on Profiles of Fatty and Amino Acids in Breast Muscles

The observed insignificant changes in SFA and MUFA concentration in breast muscles of birds fed studied microalgae diets compared to control were in agreement with previous published reports in animals [42,47,48]. On the other hand, the detected significant increase in the levels of EPA, DHA, n-3 FA, PUFA, and arachidonic acid in breast muscle of broilers chickens fed SP and AC compared to those of birds fed CV and control birds was consistent with and supported by the previous published data in animals [49] and broiler chickens [50,51]. The analysis of fatty acids of studied microalgae indicated that the fatty acid profile in the microalgae differed than that estimated in meat (Table 1). In this context, CV was rich in ⱷ-3 fatty acids than that of SP and AC. However, these fatty acids were higher in the meat of birds fed SP and AC supplemented diets than that of birds fed CV supplemented diet. Furthermore, the analysis of amino acids of studied microalgae indicated higher methionine and lysine concentrations in SP and AC than that of CV (Table 2). Additionally, high contents of methionine and lysine in SP and AC microalgae reflected positively on their levels in meat of birds fed SP and AC supplemented diets than that of birds fed CV supplemented diet. Methionine increases the concentration of total ⱷ-3 fatty acids in the breast muscle of broiler chicks [52]. In addition, lysine enhances the accumulation of ⱷ-3 fatty acids in meat of broiler chickens [52]. Therefore, the high level of n-3 fatty acids in meat of birds fed a diet supplemented with either SP or AC may be attributed to their high methionine and lysine contents compared to that of CV. In addition, further future studies are essential to confirm the mechanism by which SP and AC increase the ⱷ-3 fatty acids in broiler meat. Focused the on above discussed results, it can be said that AC and SP were preferable to CV in increasing essential fatty acid contents in broilers meat. The estimated significant increase in the levels of essential amino acids (lysine, methionine, tryptophan, and histidine) in breast muscles of broilers chickens fed SP and AC than CV and control may be attributed to their higher contents of total proteins and essential amino acids [53]. In addition, the existence of powerful antioxidants agents in SP and AC may play a role in inhibition of amino acids oxidation, which may produce during protein degradation [54]. Based on the discussion of meat amino acid profile axis, it can be said that AC and SP favorably increased the essential amino acid contents in broilers meat over CV and control.

### 4.3. Effect of Dietary Microalgae on Antioxidant Status of Breast Muscles

MDA was documented as indicator of lipid peroxidation [55]. The present findings revealed a significant decrease in MDA values in muscle tissue of birds supplemented with all species of microalgae (CV, SP, and AC) compared to the control. This finding is in agreement with previous works describing the high antioxidant capacity of SP in broiler chickens [56] which was attributed to its contents of beta-carotene zeaxanthin, phycocyanin, and allophycocyanin [57]. In addition, the higher concentration of β-carotene, fucoxanthin, and phenolic compounds of antioxidant activity in AC has been described earlier [58]. Furthermore, CV extract played a role in lowering lipid peroxidation in rats exposed to naphthalene toxicity [59]. Superoxide dismutase (SOD) is the most essential antioxidant enzyme, which plays a vital role in the removal of superoxide anion [60] in animals. The present finding of the significant increase in SOD values in muscle tissue of birds supplemented with all species of microalgae (CV, SP, and AC) compared to the control may indicate that there was a great correlation between addition of microalgae and improvement of antioxidant capacity as previously reported [56]. It was reported that the reactive oxygen species, which may produce during lipid peroxidation, has the ability to oxidize the protein side chains [61]. Thereby, any condition influencing lipid peroxidation may also lead to protein oxidation [62]. In chickens, the degree of protein oxidation was estimated by carbonyl content which is related to lipid oxidation [62]. As observed in the current study, protein carbonyl (PC) levels were decreased significantly in breast meat of chickens supplemented with all species of microalgae compared to control. This effect may be attributed to the higher antioxidant contents of microalgae [56] which may prevent oxidation of protein and free amino acids into PC. Based on the discussion of meat antioxidant status axis, it can be said that all microalgae species improved the antioxidant status in the breast muscle of broiler chickens with an equal efficiency compared to the control.

### 4.4. Effect of Dietary Microalgae on Meat Quality Parameters of Breast Muscles 

The detected reduced level of APC 1st day and APC 5th days in breast muscle of broiler chickens fed microalgae compared to the control indicated their antimicrobial activities that attributed to high contents of bioactive antimicrobial and antioxidant peptides [58,63,64]. Additionally, the preservative activity of SP and AC was stronger than that of CV which remained comparable to the control as reflected on estimated APC’s growing volumes. High levels of bioactive and antioxidant peptides [58,63,64] or eicosapentaenoic acids (EPA) may be correlated with antimicrobial capacity, particularly EPA because it is significantly higher in SP and AC than in control and CV groups. SP had higher antioxidant activity than CV, which may also contribute to a higher preservative effect of SP than CV [65]. Nevertheless, the exact compound and mechanism of action remain unknown [66]. Based on the discussion of meat quality axis, it can be said that all supplemented microalgae species, notably SP and AC, improved the technological (losses in thawing and cooking) and keeping qualities of broiler chickens meat compared to the control. In general and based on the whole discussion section, the three studied species of microalgae improved the antioxidant status of breast muscle in broiler chickens. However, AC shared CV in the superiority for growth promotion in broiler chickens. Furthermore, AC shared SP in the superiority for increment of the essential fatty and amino acids concentrations in the breast meat of broiler chickens. AC shared SP in the superiority of reduction of microbial growth in the breast muscle of broilers chickens compared to CV which remained comparable to the control as reflected on estimated APC’s growing volumes.

## 5. Conclusions

The current results may recommend supplementation of the studied microalgae species to broilers chickens’ diet for improvement of performance parameters, profiles of fatty and amino acids, antioxidant status, and meat quality. The current study may arrange the superiority of studied microalgae into *Amphora coffeaformis* (AC) > *Spirulina platensis* (SP) > *Chlorella vulgaris* (CV) as feed additives in broiler chickens. However, future studies are required to evaluate the effect of combined supplementation of the studied microalgae on growth and meat quality of broiler chickens.

## Figures and Tables

**Table 1:** Ingredient composition and calculated nutrient analysis of starter (1–10 days), grower (11–25 days), and finisher (26–36 days) diets.

Ingredients	0–10 Days	11–25 Days	26–36 Days
Yellow corn grains	48.77	52	64.05
Soybean meal 44%	34.32	33	25.00
Corn gluten meal	6.75	6	3.34
Soybean oil	5.86	5.21	3.97
Limestone	1.50	1.24	1.26
Monocalcium phosphate	1.75	1.50	1.35
Choline chloride	0.10	0.10	0.10
Sodium chloride	0.35	0.35	0.35
^1^ Vitamin and mineral premix	0.35	0.35	0.35
L-lysine HCl	0.14	0.14	0.17
DL-methionine	0.11	0.11	0.06
**Nutrient specifications**			
^2^ ME Kcal/kg diet	3200	3200	3200
CP (%)	23	20	18
Ca (%)	1.00	0.9	0.77
Available P (%)	0.46	0.45	0.38
Methionine (%)	0.50	0.45	0.37
Lysine (%)	1.10	1.00	0.90

^1^ Vitamin and mineral premix supplied each kg of feeds with: Vitamin A 12000 IU; vitamin D_3_ 2000 IU; vitamin E 10 mg; vitamin K_3_ 2 mg; vitamin B_1_ 1 mg; vitamin B_2_ 5 mg; vitamin B_6_ 1.5 mg; vitamin B_12_ 0.01 mg; Biotin 0.05 mg; pantothenic acid 10 mg; Nicotinic acid 30 mg; Folic acid 1 mg; Manganese 60 mg; Iron 30 mg; Copper 10 mg; Iodine 1 mg; Selenium 0.01 mg; Cobalt 0.01 mg. ^2^ ME: metabolizable energy.

**Table 2:** Concentrations of fatty acids (mg/g dried powder) in studied microalgae (*Spirulina platensis* (SP), *Chlorella vulgaris* (CV), and *Amphora coffeaformis* (AC)).

Fatty Acid	SP	CV	AC
Myristic (C14:0)	3.15	6.91	5.18
Plamitic (C16:0)	26.91	59.85	40.17
Stearic (C18:0)	6.49	15.27	10.74
Palmitoleic (C16:1)	1.61	3.52	2.68
Oleic (C18:1)	2.44	6.36	3.88
Linoleic (C18:2n-6)	14.30	26.37	22.47
α-Linolenic (C18:3 n-3)	4.46	11.82	6.94
Archidic (C20:0)	13.66	26.22	21.57
EPA(C20:5 n-3)	0.27	1.26	0.40
DHA (C22:6 n-3)	1.09	1.987	2.98

CV: *Chlorella vulgaris*; SP: *Spirulina platensis*; AC: *Amphora coffeaformis*.

**Table 3:** Concentrations of amino acids (mg/g dried powder) in studied microalgae (SP, CV, and AC).

Fatty Acid	SP	CV	AC
Alanine	55.17	46.70	59.37
Arginine	55.54	42.43	44.41
Aspartic acid	62.07	47.35	57.99
Glutamic acid	78.50	62.11	74.21
Glycine	36.67	33.07	36.27
Histidine	28.84	22.21	20.57
Isoleucine	17.89	15.11	14.30
Leucine	42.27	33.56	30.67
Lysine	25.30	21.97	28.14
Methionine	0.32	ND	0.30
Phenyl alanine	24.90	23.03	21.07
Serine	31.98	26.07	25.71
Taurine	1.86	1.68	ND
Threonine	29.50	26.81	30.53
Tyrosine	2.62	ND	0.78
Valine	28.16	22.88	27.68

CV: *Chlorella vulgaris*; SP: *Spirulina platensis*; AC: *Amphora coffeaformis*.

**Table 4:** Growth performance parameters of broilers chickens fed different species of microalgae for 32 days.

Parameters	Dietary Treatment				SEM	*p* Values
	Group 1	Group 2	Group 3	Group 4		
Body weight at 4 days (g)	74.27	72.56	73.83	73.50	0.43	0.61
Body weight at 36 days (g)	1844 ^b^	1990 ^a^	1862.8 ^b^	1980.5 ^a^	20.27	0.01
Body weight gain (g)	1770 ^b^	1916 ^a^	1788 ^b^	1907 ^a^	20.38	0.01
Feed intake (g)	3485	3517	3461	3500	14.38	0.32
Feed conversion ratio	1.97	1.84	1.93	1.84	0.14	0.05

^a–b^ Means within a row not sharing a common superscript differ significantly when (*p* < 0.05). SEM: standard error of mean; Group 1: control; Group 2: *Chlorella vulgaris* (CV)-supplemented group; Group 3: *Spirulina platensis* (SP)-supplemented group; Group 4: *Amphora coffeaformis* (AC)-supplemented group.

**Table 5:** Profiles of fatty acids in breast muscle of broilers chickens fed different species of microalgae for 32 days.

Fatty Acids	Dietary Treatment				SEM	*p* Values
	Group 1	Group 2	Group 3	Group 4		
Myristic (C14:0)	0.71	0.70	0.70	0.70	0.01	0.98
Plamitic (C16:0)	19.41	18.99	19.45	21.02	0.41	0.35
Stearic (C18:0)	7.26	7.54	7.55	7.53	0.14	0.91
Archidic (C20:0)	0.92	0.88	0.89	0.95	0.02	0.76
Palmitoleic (C16:1)	3.18	3.19	3.40	3.21	0.06	0.64
Oleic (C18:1)	20.59	20.31	20.63	18.82	0.47	0.52
Linoleic (C18:2n-6)	18.29	17.11	16.85	18.41	0.36	0.33
α-Linolenic (C18:3 n-3)	1.71	1.73	1.57	1.71	0.03	0.32
Stearidonic acid (C18:4n3)	0.92	0.89	0.85	0.97	0.01	0.162
EPA(C20:5 n-3)	0.91 ^b^	0.97 ^b^	2.19 ^a^	1.69 ^a^	0.15	0.001
DHA (C22:6 n-3)	1.11 ^b^	1.19 ^b^	2.40 ^a^	2.20 ^a^	0.15	< 0.001
Arachidonic acid(C20:4n6)	1.04 ^b^	1.21 ^b^	2.56 ^a^	2.25 ^a^	0.17	< 0.001
Total SFA	28.29	28.03	28.61	30.31	0.45	0.31
MUFA	23.78	23.51	24.04	22.03	0.47	0.49
PUFA	23.93 ^b^	23.13 ^b^	26.45 ^a^	27.7 ^a^	0.58	0.003
PUFA/SFA	0.84	0.82	0.92	0.91	0.02	0.24
Total n-3 FA	4.59 ^b^	4.80 ^b^	7.02 ^a^	6.84 ^a^	0.29	< 0.001

^a–b^ Means within a row not sharing a common superscript differ significantly when (*p* < 0.05). SEM: standard error of mean; EPA: Eicosapentaenoic; DHA: Docosahexaenoic; SFA: saturated fatty acids; MUFA: monounsaturated fatty acids; PUFA: polyunsaturated fatty acids; n-3 FA: omega-3 fatty acids; Group 1: control; Group 2: *Chlorella vulgaris* (CV)-supplemented group; Group 3: *Spirulina platensis* (SP)-supplemented group; Group 4: *Amphora coffeaformis* (AC)-supplemented group.

**Table 6:** Profiles of amino acids in breast muscle of broilers chickens fed different species of microalgae for 32 days.

Amino Acids	Dietary Treatment				SEM	*p* Values
	Group 1	Group 2	Group 3	Group 4		
Lysine	6.04 ^b^	6.39 ^b^	7.18 ^a^	7.02 ^a^	0.15	0.009
Leucine	6.80	6.73	7.63	7.55	0.16	0.09
Isoleucine	3.72	3.86	4.40	3.59	0.11	0.06
Valine	4.82	4.51	4.91	4.32	0.11	0.27
Methionine	1.97 ^b^	1.87 ^b^	2.33 ^a^	2.23 ^a^	0.06	0.04
Phenyl alanine	2.10	2.15	2.51	2.29	0.07	0.16
Tryptophan	2.53 ^b^	2.50 ^b^	2.92 ^a^	2.86 ^a^	0.07	0.002
Threonine	3.04	3.03	3.51	3.13	0.09	0.24
Histidine	3.04 ^b^	3.18 ^b^	3.82 ^a^	3.75 ^a^	0.10	0.02
Arginine	5.47	4.98	5.66	5.29	0.12	0.26
Glycine	4.48	4.05	4.28	4.22	0.10	0.56
Proline	1.68	1.72	1.51	1.61	0.03	0.12
Serine	2.71	2.86	2.58	2.78	0.07	0.60
Aspartic acid	8.28 ^b^	8.38 ^b^	9.38 ^a^	9.73 ^a^	0.18	0.01
Glutamic acid	15.96	16.77	16.58	16.26	0.69	0.12
Alanine	5.26	5.32	5.35	5.06	0.12	0.88

^a–b^ Means within a row not sharing a common superscript differ significantly when (*p* < 0.05). SEM: standard error of mean; Group 1: control; Group 2: *Chlorella vulgaris* (CV)-supplemented group; Group 3: *Spirulina platensis* (SP)-supplemented group; Group 4: *Amphora coffeaformis* (AC)-supplemented group.

**Table 7:** Antioxidant status in breast muscle of broilers chickens fed different species of microalgae for 32 days.

Muscle Tissue	Dietary Treatment				SEM	*p* Values
	Group 1	Group 2	Group 3	Group 4		
MDA (nmol/g tissue)	19.76 ^a^	14.32 ^b^	13.31 ^b^	13.61 ^b^	0.62	0.007
SOD (U/g tissue)	36.94 ^b^	55.08 ^a^	60.70 ^a^	53.39 ^a^	3.88	0.02
PC (nmol/mg protein)	33.66 ^a^	26.55 ^b^	25.41 ^b^	26.10 ^b^	1.18	0.01

^a–b^ Means within a row not sharing a common superscript differ significantly when (*p* < 0.05). SEM: standard error of mean; MDA: malondialdehyde; PC: protein carbonyl; SOD: superoxide dismutase; Group 1: control; Group 2: *Chlorella vulgaris* (CV)-supplemented group; Group 3: *Spirulina platensis* (SP)-supplemented group; Group 4: *Amphora coffeaformis* (AC) supplemented group.

**Table 8:** Meat quality parameters in breast muscle of broilers chickens fed different species of microalgae for 32 days.

Parameters	Dietary Treatment				SEM	*p* Values
	Group 1	Group 2	Group 3	Group 4		
pH24	5.74	5.86	5.93	5.81	0.03	0.29
Water holding capacity (%)	73.70	73.49	78.86	69.79	2.09	0.59
Thawing loss (%)	5.06	5.20	4.77	4.06	0.70	0.96
Cooking loss (%)	18.63 ^a^	12.56 ^b^	12.39 ^b^	11.69 ^b^	1.01	0.02
APC 1st day (log cfu/gm)	5.37 ^a^	4.30 ^b^	4.62 ^b^	4.93 ^b^	0.13	0.002
APC 5th day (log cfu/gm)	6.54 ^a^	5.50 ^b^	5.59 ^b^	5.79 ^b^	0.14	< 0.001
APC’s growing volumes (log cfu/gm)	1.17 ^a^	1.20 ^a^	0.97 ^b^	0.85 ^b^	0.12	0.098

^a–b^ Means within a row not sharing a common superscript differ significantly when (*p* < 0.05). SEM: standard error of mean; PH_24_: meat PH after 24 h; aerobic plate count (APC) 1st day: aerobic plate count at first day; APC 5th day: aerobic plate count after five days; Group 1: control; Group 2: *Chlorella vulgaris* (CV)-supplemented group; Group 3: *Spirulina platensis* (SP)-supplemented group; Group 4: *Amphora coffeaformis* (AC)-supplemented group. APC’s growing volumes is the difference between APC 5th day and APC 1st day.
